# Antiviral effect of cetylpyridinium chloride in mouthwash on SARS-CoV-2

**DOI:** 10.1038/s41598-022-18367-6

**Published:** 2022-08-18

**Authors:** Ryo Takeda, Hirofumi Sawa, Michihito Sasaki, Yasuko Orba, Nako Maishi, Takuya Tsumita, Natsumi Ushijima, Yasuhiro Hida, Hidehiko Sano, Yoshimasa Kitagawa, Kyoko Hida

**Affiliations:** 1 Vascular Biology and Molecular Pathology, Faculty of Dental Medicine and Graduate School of Dental Medicine, Hokkaido University, Sapporo, Japan; 2 Oral Diagnosis and Medicine, Faculty of Dental Medicine and Graduate School of Dental Medicine, Hokkaido University, Sapporo, Japan; 3 Division of Molecular Pathobiology, International Institute for Zoonosis Control, Hokkaido University, Sapporo, Japan; 4 International Collaboration Unit, International Institute for Zoonosis Control, Hokkaido University, Sapporo, Japan; 5 One Health Research Center, Hokkaido University, Sapporo, Japan; 6 Support Section for Education and Research, Graduate School of Dental Medicine, Hokkaido University, Sapporo, Japan; 7 Community Service and Welfare Network, Hokkaido University Hospital, Sapporo, Japan; 8 Restorative Dentistry, Faculty of Dental Medicine and Graduate School of Dental Medicine, Hokkaido University, Sapporo, Japan

**Keywords:** Infectious diseases, Virology

## Abstract

Cetylpyridinium chloride (CPC), a quaternary ammonium compound, which is present in mouthwash, is effective against bacteria, fungi, and enveloped viruses. This study was conducted to explore the antiviral effect of CPC on SARS-CoV-2. There are few reports on the effect of CPC against wild-type SARS-CoV-2 at low concentrations such as 0.001%–0.005% (10–50 µg/mL). Interestingly, we found that low concentrations of CPC suppressed the infectivity of human isolated SARS-CoV-2 strains (Wuhan, Alpha, Beta, and Gamma) even in saliva. Furthermore, we demonstrated that CPC shows anti-SARS-CoV-2 effects without disrupting the virus envelope, using sucrose density analysis and electron microscopic examination. In conclusion, this study provided experimental evidence that CPC may inhibit SARS-CoV-2 infection even at lower concentrations.

## Introduction

According to the recent information from the coronavirus resource center, Johns Hopkins University of Medicine^1^, COVID-19 is responsible for more than 420 million cases and around 6 million deaths worldwide.

SARS-CoV-2 was originally reported in Wuhan, China^2^ and some variants of interest and variants of concern (VOCs) have also been reported^3^. In addition, it is concerned that some variants like Delta and Omicron might have the ability to evade vaccine-induced immunity^4–6^. Therefore, scientists concern that SARS-CoV-2 pandemic may continue even after the increase in vaccination coverage.

It has been reported that SARS-CoV-2 infects epithelial cells of oral mucosa and salivary glands, which express viral entry factors, angiotensin-converting enzyme 2 (ACE2), and the trans- membrane protease serine (TMPRSS) members^7^. Thus, in this fashion oral cavity plays a crucial role in infection and transmission of SARS-CoV-2. Although the symptom of COVID-19 related to oral cavity is dysgeusia and stomatitis^8,9^, many SARS-CoV-2-infected people could be asymptomatic, resulting in its transmission to other people.

SARS-CoV-2 can replicate in oral cavity and release into saliva^7^. In addition, SARS-CoV-2 can replicate in respiratory epithelium^10^ and may be transmitted to oral cavity by coughing. Transmission of SARS-CoV-2 through droplets and/or aerosol causes its infection and replication in lung alveolar epithelial cells, resulting in alveolar damage^11^. Furthermore, it is reported that SARS-CoV-2 transmission occurs through droplets from expiratory activities, such as talking, coughing, and sneezing^12,13^. Interestingly, the SARS-CoV-2 infected people may become a source of transmission even during the asymptomatic incubation period of the virus^14^. Thus, we need to investigate the prophylaxis strategy against COVID-19. Furthermore, the relationship between aspiration of droplets from saliva containing SARS-CoV-2 and COVID-19 aggravation has been reported^15^. Therefore, oral care is important for prevention of transmission of SARS-CoV-2.

Mouthwash has been focused on preventing microbiome infection^16^. In addition, several components of mouthwash have recently been reported to reduce SARS-CoV-2 virions in the oral cavity^17,18^. Cetylpyridinium chloride (CPC) is widely used as one of the bactericidal components of mouthwash, tablets, sprays, and drops. CPC can disrupt the lipid membrane through physicochemical interactions. CPC has already been reported to have bactericidal effects as well as antiviral effects against influenza virus^19^ and coronaviruses^20–22^. Compared to other ingredients in mouthwashes, including povidone iodine and chlorhexidine (CHX); CPC is tasteless, odourless, and thus suitable for applications in oral care products. To date, there are few reports depicting virucidal activity of CPC against SARS-CoV-2. Seneviratne et al. reported that CPC reduced viral load of SARS-CoV-2 in the saliva of four patients with COVID-19^23^ compared to control water, but the viral infectivity in saliva was not described. Recent report showed the effect of CPC at much lower concentration than that of CPC in commercially available mouthwashes against pseudovirus^24^. But there is no report on the effect of CPC at low concentrations such as 0.001%–0.005% (10–50 µg/mL) against wild-type SARS-CoV-2 in saliva. In Japan, the concentration of CPC in commercially available mouthwashes is almost 30–50 µg/mL, which is much lower than in the mouthwashes used in the previous reports^24,25^. Therefore, we examined the antiviral effects of CPC on SARS-CoV-2 at low concentrations. In addition, we also examined the mechanism of CPC’s anti-SARS-CoV-2 activity by sucrose density analysis and electron microscopical observation.

## Results

### CPC suppressed SARS-CoV-2 infectivity

**Figure 1:**
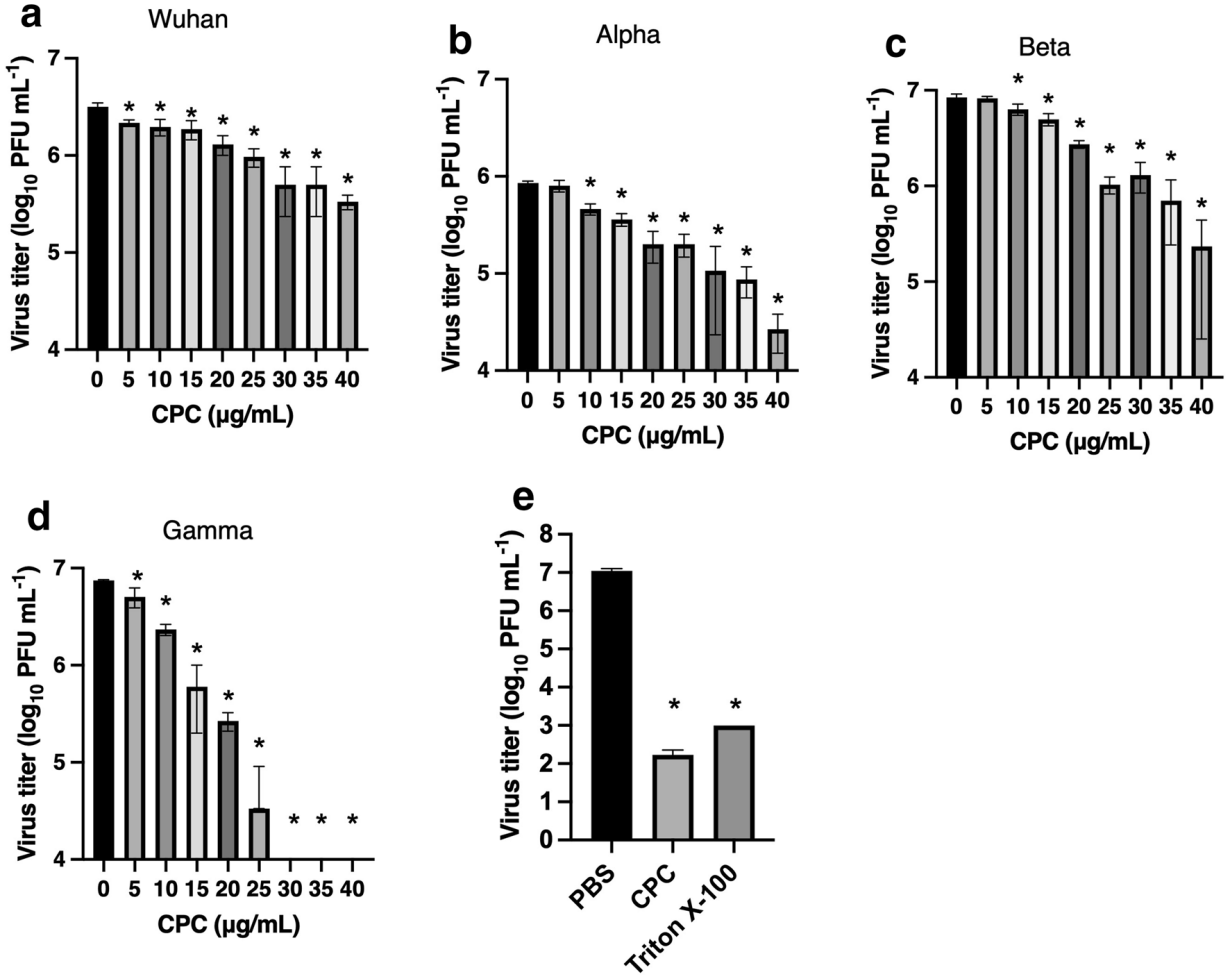
Antiviral efficacy of CPC against SARS-CoV-2 by plaque assay using Vero E6 cells expressing the TMPRSS2 gene (VeroE6/TMPRSS2). The virus titers were counted and the virus titer of SARS-CoV-2 Wuhan (**a**), Alpha (**b**), Beta (**c**) and Gamma (**d**) strains treated by CPC (0–40 μg/mL) at room temperature for 30 min were quantified and represented as PFU/mL. Plaque assay was also performed in the presence of PBS, CPC (50 μg/mL) or Triton X-100 (1%) for 10 min. Thereafter, samples were filtered by PD-10 columns to eliminate reagents (**e**). Statistical analysis was performed using one-way analysis of variance. (*p < 0.05).

We have examined the SARS-CoV-2 strains, including Wuhan, Alpha, Beta, and Gamma, which belong to VOC. The plaque assay demonstrated that CPC significantly suppressed the infectivity of all examined SARS-CoV-2 directly in a dose-dependent manner (Figs. 1a–d, S1). CPC (50 μg/mL) treatment completely inactivated SARS-CoV-2 Wuhan strain similarly as Triton X-100 (1%) (Fig. 1e). A commercial mouthwash (SP-T medical gargle: SP-T) containing the same concentration of CPC showed a better antiviral effect than CPC solution with no interfering ingredients. The virus titer of SARS-CoV-2 treated with SP-T was below the limit of detection of 2.0 × 10^3^ PFU/mL (Fig. S2). These results indicated that the lower concentrations of CPC (10–40 μg/mL) than those of commercially available mouthwash (50 μg/mL) exhibited anti-SARS-CoV-2 effects in many strains, including VOC.

**Figure 2:**
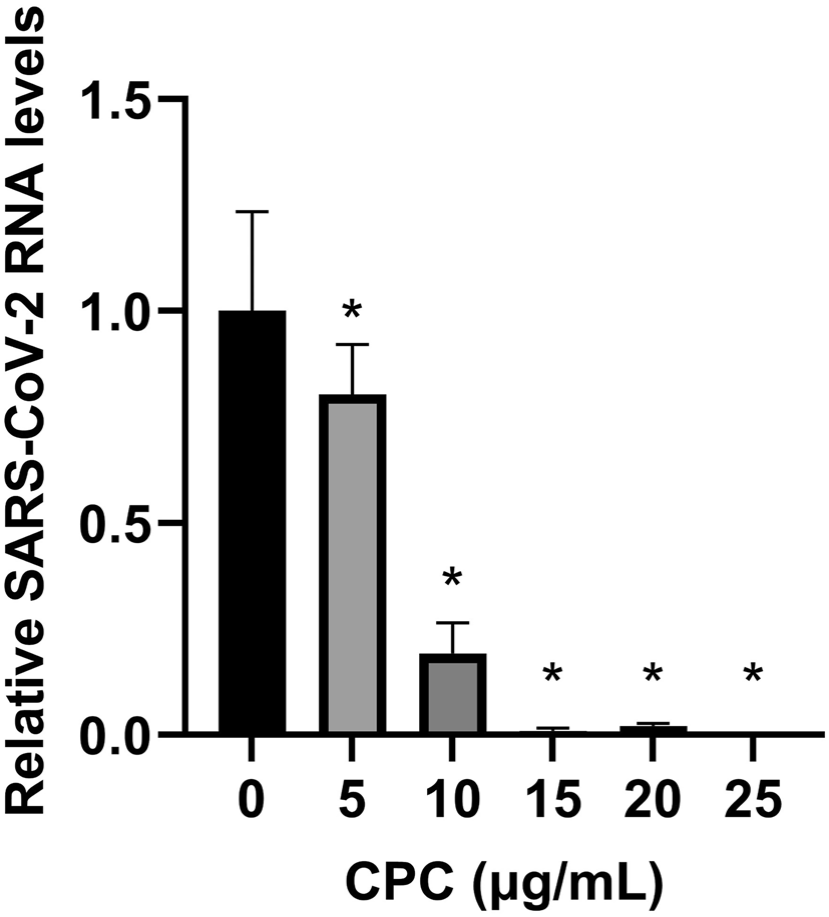
Antiviral efficacy of CPC against SARS-CoV-2 by qRT-PCR. VeroE6/TMPRSS2 cells were inoculated with SARS-CoV-2 Wuhan strain at a multiplicity of infection (MOI) of 0.01 after mixing equal amount CPC. At 24 h postinfection, the relative levels of viral N protein RNA were evaluated quantitatively by qRT-PCR. (*p < 0.05).

Next, we assessed effect of CPC on cell entry of SARS-CoV-2. The VeroE6/TMPRSS2 cells were infected with CPC-treated SARS-CoV-2 Wuhan strain at multiplicity of infection (MOI) of 0.01. Viral RNA expression level in the cells was significantly reduced by CPC via dose-dependent manner at 24 h postinfection (Fig. 2). The viral RNA copy number was reduced to around one-thirtieth by CPC at the concentration of 15 μg/mL compared to control. These data indicated that the amounts of infectious virions were decreased by CPC before cell entry. All experiments have been performed using CPC at the concentration which did not cause cytotoxicity (Fig. S3).

### CPC has antiviral activity against SARS-CoV-2 even in saliva

**Figure 3:**
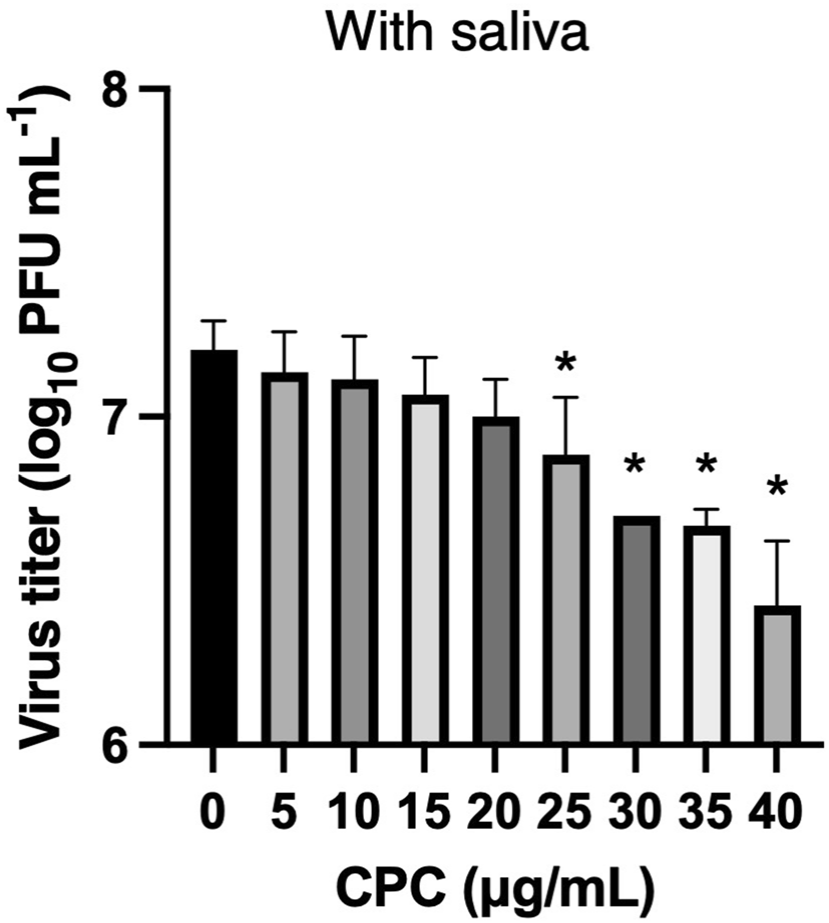
Antiviral efficacy of CPC against SARS-CoV-2 with saliva by plaque assay using Vero E6 cells expressing the TMPRSS2 gene (VeroE6/TMPRSS2). SARS-CoV-2 Wuhan strain was added in saliva and mixed with equal amount CPC. (*p < 0.05).

To address whether CPC is effective on SARS-CoV-2 in saliva that contains many proteins and is highly viscous, we measured infectivity of SARS-CoV-2 Wuhan strain by plaque assay after incubation with CPC in saliva collected from healthy volunteers. Plaque assay demonstrated the inhibitory effect of CPC (25–40 μg/mL) against SARS-CoV-2 in saliva significantly in a dose-dependent manner (Fig. 3).

### Sucrose density analysis and transmission electron microscopy analysis of SARS-CoV-2 treated with CPC

**Figure 4:**
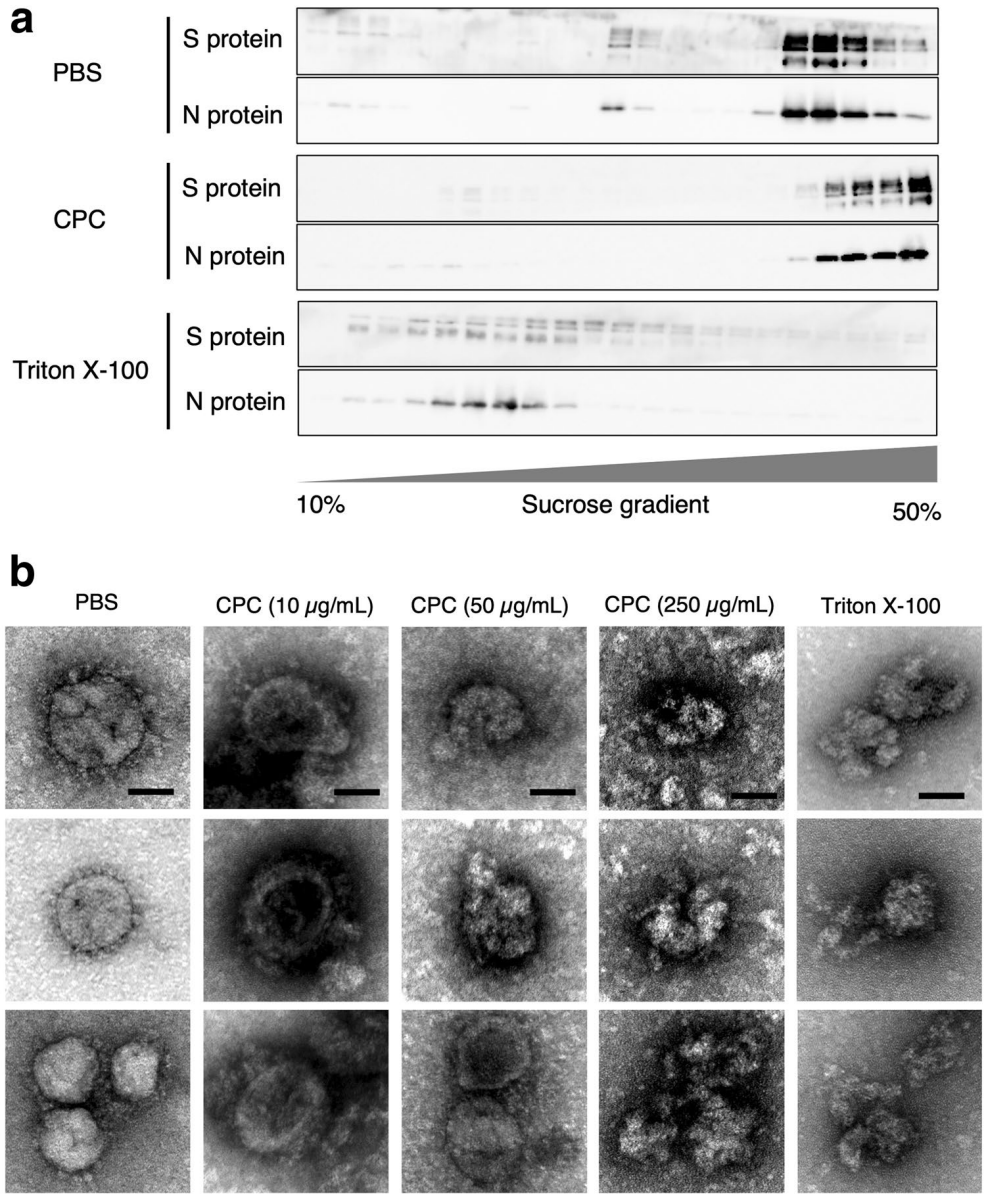
Sucrose density analysis and TEM analysis of SARS-CoV-2 particles. (**a**) Sucrose density analysis of capsid assembly in the presence of 1 × PBS, CPC (50 μg/mL) and Triton X-100 (1%). SARS-CoV-2 Wuhan strain was treated with described regents for 10 min, and the treated virions were applied to the density-gradient ultracentrifugation. Each fraction was applied to SDS-PAGE and analyzed by Western blotting with antibodies against S protein and N protein. (**b**) Electron micrographs of SARS-CoV-2 virions after treatment with reagents. SARS-CoV-2 Wuhan strain was treated with 1 × PBS, CPC (10, 50, 250 μg/mL) and Triton X-100 (1%) for 10 min at room temperature. Each scale bar represents 50 nm.

To analyze the mechanism of CPC on SARS-CoV-2 infectivity, we performed sucrose density analysis of SARS-CoV-2 virions treated by either 1 × phosphate-buffered saline (PBS), CPC (50 μg/mL) or Triton X-100 (1%) for 10 min at room temperature (Fig. 4a). SARS-CoV-2 S and N protein showed a specific distribution throughout the gradient. The band shift was observed in the virions treated with Triton X-100; however, the fractions were different than those treated with PBS or CPC. In other words, PBS and CPC might have no effect on the structure of the SARS-CoV-2 virions, whereas Triton X-100 changed the structure. Furthermore, the morphology of virions was analyzed by transmission electron microscopy (TEM). Electron microscopical analysis revealed that the spherical particle structure of SARS-CoV-2 treated with PBS remained unchanged. We also found that most virus particles treated with 10 µg/mL CPC remained unchanged, whereas some disintegrated with 50 µg/mL CPC. In contrast, almost all virus particles treated with 250 µg/mL CPC were clearly disrupted (like 1% Triton X-100). The amount of 50 µg/mL was considered a concentration at which all virus particles did not shatter. This result is consistent with sucrose density analysis data (Fig. 4b).

## Discussion

In this research, we demonstrated that CPC at low concentration 50 μg/mL or less, suppresses infectivity of SARS-CoV-2 Wuhan strain and VOCs, including Alpha, Beta, and Gamma strains. CPC showed virucidal effects against SARS-CoV-2 even in saliva. Thus, we found that low concentrations of CPC exerted sufficient antiviral activity against SARS-CoV-2. Because CPC disrupts lipid bilayers, high concentrations of CPC may exert cytotoxic effects. Therefore, CPC can be applied in a formulation that can exert its effect for a long time at low concentrations. Because the prototype mouthwash formulation may contain other interfering ingredients to quench the low levels of CPC used in the experiments, we tested a commercial mouthwash with the same concentration of CPC, which showed the same or better antiviral effect than the CPC solution without interfering ingredients (Fig. S2). Furthermore, we could suggest that the antiviral effect may not be due to destruction of the lipid membrane but due to SARS-CoV-2 protein denaturation.

It has been reported that CPC is effective against SARS-CoV-2 Alpha variant^24^. Our study revealed that CPC attenuates the infectivity of two more variants (Beta and Gamma strains) at low concentrations. CPC treatment for 1 h did not show the cytotoxicity to VeroE6/TMPRSS2 up to 40 μg/mL (Fig. S3). Furthermore, in previous studies, mouthwash containing higher concentration of CPC was used (500–750 μg/mL)^24,25^. At these concentrations, it is considered that the lipid membrane of cells might be damaged.

The duration of the antiviral effect of mouthwash containing CPC has not yet been clear; however, our results suggest that a lower concentration is enough to show antiviral effect. As a limitation of this experiment, when CPC is applied as a mouthwash, the duration of action is assumed to be within a minute. However, considering the complexity of the experimental technique and possibility of errors in the duration of action among samples, the minimum duration of action was set to 10 min in our study. In this regard, Anderson et al. have clarified the effect of CPC at an action time of 30 s using a method to neutralize CPC^26^. Thus, it is warranted to develop new products, such as tablets, drops, and patches that can release CPC at a safe concentration and can be retained in oral cavity for as long as possible.

We showed that CPC suppressed SARS-CoV-2 infectivity in viscous saliva, which contains various proteins. However, it seems that the effect is weakened in saliva compared to PBS. This may be due to the presence of negatively charged saliva proteins, decreased diffusion efficiency due to viscosity and micelle formation^27^. However, even low concentrations of CPC showed sufficient suppression of SARS-CoV-2 infectivity. The suppressive effects low concentrations of CPC on infectivity of SARS-CoV-2 in saliva of actual COVID-19 patients remain to be elucidated.

The mechanism of action that suppresses the infectivity of SARS-CoV-2 has been thought to be due to the destruction of the viral envelope. This study showed that CPC inactivates SARS-CoV-2 without disrupting the viral particle at the concentration and duration of the experiments. It has been reported high concentration (250–500 μg/mL) of CPC disrupts the envelope of SARS-CoV-2^22,28^. Thus, the CPC concentration–dependent degree of morphological breakdown varies. In other words, the particles are crumpled, but not shattered, at a concentration that is still sufficient to deactivate the virus. These results are consistent with the results of the sucrose density-gradient analysis. Additionally, for the above reasons, the antiviral mechanism of CPC shown by our study supports the finding that CPC interferes chiefly with the lipid membrane^29^. Figure 4b shows virus particles of various sizes. In previous reports, the diameter of SARS-CoV-2 varied from approximately 60 to 140 nm, which is consistent with the present results^2,5,30^. However, the detailed mechanism of inactivation of the SARS-CoV-2 by lower concentration of CPC is still unclear. Our results suggest that the denaturing effect of the S protein may be involved in entry, and it seems to play a crucial role.

The entry of viruses into the organism is thought to be through an oral cavity and nasal cavity^31^. By applying CPC-containing nasal sprays and reducing the amount of virus in the nasal cavity, it may lead to the control of COVID-19 infection. However, to date, there is no report exhibiting the use of nasal CPC sprays and the preventive effect of them is still unknown.

Currently, we are conducting a clinical study to examine the effect of CPC in COVID-19 patients, which addresses the effect of CPC on SARS-CoV-2 viral load in patient saliva. A low viral load in saliva might result in a low transmission rate and a less progression of the disease status.

The use of CPC-containing products may lead to a reduction in the number of newly infected covid patients. Additionally, it may be a means of preventive measures in poorly vaccinated countries. We anticipate that CPC will be used as one of the tools to prevent the onset and infection of SARS-CoV-2.

## Materials and methods

### Cell culture

Vero E6 (ATCC, Manassas, VA, USA) cells were maintained in Dulbecco’s Modified Eagle’s Medium (DMEM) supplemented with 10% fetal bovine serum (FBS) (v/v) and incubated at 37 °C with 5% CO2. Vero E6 stably expressing human TMPRSS2 (VeroE6/TMPRSS2) cells^32^ were also used for this study.

### Viruses

The SARS-CoV-2 Wuhan (WK-521; EPI_ISL_408667), Alpha (QK002; EPI_ISL_768526), Beta (TY8-612; EPI_ISL_1123289), and Gamma (TY7-501; EPI_ISL_833366) strains were kindly provided by Dr. Saijo (National Institute of Infectious Diseases, Tokyo, Japan). These viruses were prepared using VeroE6/TMPRSS2 cells. All experiments using SARS-CoV-2 were performed at the Biosafety Level-3 (BSL-3) facility of the International Institute for Zoonosis Control (approved number: 19(19), #21002-3), Hokkaido University and followed the standard operating procedures of BSL-3. In addition, all experimental designs using pathogens were approved by the Graduate school of dental medicine, Hokkaido University (approved number: R-2-4-1).

### Reagents

CPC (TCI, Tokyo, Japan) was dissolved with deionized distilled water (DDW) and sterilized by a filter (0.45 µm in diameter), (Sartorius, Göttingen, Germany). In addition, Triton X-100 (Sigma-Aldrich, St. Louis, MO, USA), a surfactant, was used as a positive control. PBS was used as a negative control.

### Saliva from healthy volunteers

Saliva was provided from five healthy unvaccinated volunteers. All saliva samples were determined to be negative for SARS-CoV-2 by quantitative reverse transcription polymerase chain reaction (qRT-PCR) prior to the experiments and mixed in one tube. This experiment was approved by the Institutional Ethics Committee of Hokkaido University to use human-derived materials. Informed consent was obtained from each volunteer before collecting saliva (approved number: 2021-2).

### Cell survival assay

Cell viability of VeroE6/TMPRSS2 was measured by an MTS [3-(4,5-dimethylthylthiazol-2-yl)-5-(3-carboxymethoxyphenyl)-2-(4-sulfophenyl)-2H-tetrazolium] assay using CellTiter 96 AQueous One Solution (Promega, Madison, WI, USA) in the presence of different concentration of CPC (0–50 µg/mL) for 1 h at 37 °C. The absorbance was measured with GloMax Multiplus Plate Reader/Luminometer (Promega). Three independent experiments were performed in triplicate.

### Plaque assay

SARS-CoV-2 strains were mixed with an equal amount of CPC solution (final concentration: 0–50 µg/mL with DMEM containing 2% FBS) or SP-T medical gargle (Lion Corporation, Tokyo, Japan), which was diluted in PBS to a concentration of 50 µg/mL, similar to CPC. The mixture was incubated for 30 min at room temperature and diluted to 1/10 with 2% FBS DMEM to reduce CPC in the mixture. The diluted mixture was inoculated in VeroE6/TMPRSS2 cells and incubated at 37 °C for 1 h with rotation. After incubation, the cells were washed with 1 × PBS twice to remove CPC and were then overlaid with 2% FBS DMEM containing 1.2% Bacto Agar (Becton Dickinson, Franklin Lakes, NJ, USA). After 48 h incubation at 37 °C, cells were fixed with 3.7% buffered formaldehyde overnight. Fixed cells were stained with 1% crystal violet. Cells infected with SARS-CoV-2 demonstrated cytopathic effects, and the infected cell clusters can be seen as unstained areas, such as plaques.

### Virus entry assay

VeroE6/TMPRSS2 cells were seeded on 24-well plates at a density of 1.0 × 10^5^ cells/well. SARS-CoV-2 Wuhan strain was mixed with equal amount of CPC (Final concentration: 0–25 μg/mL). At each drug concentration, the wells were infected with 1.0 × 10^3^ PFU (MOI = 0.01) of virus. The mixtures were incubated for 30 min at room temperature. After incubation, the mixtures were inoculated into VeroE6/TMPRSS2 cells and incubated at 37 °C for 1 h with rotation. After 1 h of absorption, cells were washed twice with 1 × PBS to remove CPC and cultured in maintenance medium. At 24 h postinfection (hpi), total RNAs were extracted from inoculated cells using TRIzol™ Reagent (Thermo Fisher Scientific, Waltham, MA, USA) and total RNA was extracted with RNeasy Mini Kit (QIAGEN, Hilden, Germany). The extracted RNAs were subjected to qRT-PCR analysis with the THUNDERBIRD Probe One-step qRT-PCR Kit (TOYOBO, Osaka, Japan). The SARS-CoV-2 genome was quantified using primer probe sets for N2 (Takara, Shiga, Japan). Nonhuman primate β-actin was employed as endogenous control. The primer and probe sequences for nonhuman primate β-actin were described previously^33^. Levels of N gene of SARS-CoV-2 were normalized with that of β-actin mRNA^34^. Furthermore, viral RNA levels at 24 hpi were normalized with viral RNA levels at 0 hpi. All RT-PCR tests were carried out using the CFX96 Real-Time PCR System (BioRad, Hercules, CA, USA). Three independent experiments were performed in triplicate.

### Analysis of the virucidal activity of CPC against SARS-CoV-2 in saliva

SARS-CoV-2 Wuhan strain was added in saliva collected from healthy volunteer and mixed with equal amount of CPC (Final concentration: 0–40 μg/mL). The saliva mixtures were diluted to 1/100 to reduce viscosity and filtered through 0.45 μm filters (Sartorius) to remove bacteria and fungi. Plaque assay was performed as previously described (Section “Plaque assay”). Three independent experiments were performed in triplicate.

### Sucrose density-gradient analysis

The SARS-CoV-2 Wuhan strain was treated with CPC (50 μg/mL) or Triton X-100 (1%) at room temperature for 10 min. After incubation, the mixtures were loaded on top of 10%–50% sucrose density gradients. Following ultracentrifugation with Optima XE-90 (Beckman Coulter, Brea, CA, USA) for 6 h at 250,000×*g*, each 100 μL of gradients were fractionated into 22 fractions and mixed with 100 μL of SDS-PAGE sample buffer and boiled at 95 °C for 5 min. After boiling, they were analyzed by 10% SDS-PAGE followed by immunoblot analysis using mouse monoclonal anti-SARS-CoV-2 S protein (GTX632604) or rabbit polyclonal N protein (GTX135357) antibody (GeneTex, Irvine, CA, USA). The membranes were cut at 100 kDa after the transfer and hybridized with S protein antibody and with N protein antibody respectively and subjected to visualization. An ImageQuant LAS 4000 mini (FUJIFILM Corporation, Tokyo, Japan) was used for imaging (Fig. S4).

### Transmission electron microscopy

SARS-CoV-2 Wuhan strain was treated with 1 × PBS, CPC (10, 50, and 250 μg/mL) and Triton X-100 (1%) for 10 min at room temperature. The mixtures were fixed with 2.5% glutaraldehyde at 4 °C for 24 h. Fixed sample (5 μL) was placed onto a sheet of Parafilm. Formvar Film (#10-1009, Okenshoji, Tokyo, Japan) was placed on each drop to adsorb virus for 5 min. Grids were washed with DDW and placed on a drop of filtered 2.0% uranyl acetate solution for an additional 1 min, air dried, and examined using a JEM-1400 TEM (JEOL, Tokyo, Japan) at 80 kV.

### Statistics

Statistical analyses were performed using Graphpad Prism v9 (GraphPad Software Inc., San Diego, CA, USA). Data is presented as the mean values ± S.D. of biological triplicates. Statistical analysis was performed using one-way analysis of variance. For all data sets, a *p* value of less than 0.05 was considered significant.

### Institutional review board

The study was conducted in accordance with the Declaration of Helsinki and approved by the Institutional Ethics Committee of Hokkaido University (approved number: 2021–2).

### Informed consent

Informed consent was obtained from all subjects involved in the study.

## Supplementary Information

Supplementary Figures.

## Data Availability

The datasets generated during and/or analyzed during the current study are available from the corresponding author on reasonable request.

## References

[1] COVID-19 Dashboard. (2022). https://coronavirus.jhu.edu/map.html.

[2] Zhu N (2020). A novel coronavirus from patients with pneumonia in China, 2019. N. Engl. J. Med..

[3] Tracking SARS-CoV-2 variants. (2022). https://www.who.int/en/activities/tracking-SARS-CoV-2-variants/.

[4] Bergwerk M (2021). Covid-19 breakthrough infections in vaccinated health care workers. N. Engl. J. Med..

[5] Karim SSA, Karim QA (2021). Omicron SARS-CoV-2 variant: A new chapter in the COVID-19 pandemic. Lancet.

[6] Lopez Bernal J (2021). Effectiveness of covid-19 vaccines against the B.1.617.2 (delta) variant. N. Engl. J. Med..

[7] Huang N (2021). SARS-CoV-2 infection of the oral cavity and saliva. Nat. Med..

[8] Aziz M (2020). Taste changes (dysgeusia) in COVID-19: A systematic review and meta-analysis. Gastroenterology.

[9] Iranmanesh B, Khalili M, Amiri R, Zartab H, Aflatoonian M (2021). Oral manifestations of COVID-19 disease: A review article. Dermatol. Ther..

[10] Milewska A (2020). Replication of severe acute respiratory syndrome coronavirus 2 in human respiratory epithelium. J. Virol..

[11] Schaefer I-M (2020). In situ detection of SARS-CoV-2 in lungs and airways of patients with COVID-19. Mod. Pathol..

[12] Adam DC (2020). Clustering and superspreading potential of SARS-CoV-2 infections in Hong Kong. Nat. Med..

[13] Wang Chia C (2021). Airborne transmission of respiratory viruses. Science.

[14] Lauer SA (2020). The incubation period of coronavirus disease 2019 (COVID-19) from publicly reported confirmed cases: Estimation and application. Ann. Intern. Med..

[15] Wölfel R (2020). Virological assessment of hospitalized patients with COVID-2019. Nature.

[16] Eggers M, Koburger-Janssen T, Eickmann M, Zorn J (2018). In vitro bactericidal and virucidal efficacy of povidone-iodine gargle/mouthwash against respiratory and oral tract pathogens. Infect. Dis. Ther..

[17] Garcia-Sanchez A (2022). Povidone-iodine as a pre-procedural mouthwash to reduce the salivary viral load of SARS-CoV-2: A systematic review of randomized controlled trials. Int. J. Environ. Res. Public Health.

[18] Garcia-Sanchez A (2022). Virucidal activity of different mouthwashes against the salivary load of SARS-CoV-2: A narrative review. Healthcare.

[19] Popkin DL (2017). Cetylpyridinium chloride (CPC) exhibits potent, rapid activity against influenza viruses in vitro and in vivo. Pathog. Immun..

[20] Shen L (2019). High-throughput screening and identification of potent broad-spectrum inhibitors of coronaviruses. J. Virol..

[21] Buonavoglia A (2021). Antibiotics or no antibiotics, that is the question: An update on efficient and effective use of antibiotics in dental practice. Antibiotics.

[22] Bañó-Polo M (2022). Cetylpyridinium chloride promotes disaggregation of SARS-CoV-2 virus-like particles. J. Oral Microbiol..

[23] Seneviratne CJ (2021). Efficacy of commercial mouth-rinses on SARS-CoV-2 viral load in saliva: Randomized control trial in Singapore. Infection.

[24] Muñoz-Basagoiti J (2021). Mouthwashes with CPC reduce the infectivity of SARS-CoV-2 variants in vitro. J. Dent. Res..

[25] Komine A, Yamaguchi E, Okamoto N, Yamamoto K (2021). Virucidal activity of oral care products against SARS-CoV-2 in vitro. J. Oral Maxillofac. Surg. Med. Pathol..

[26] Anderson ER (2022). CPC-containing oral rinses inactivate SARS-CoV-2 variants and are active in the presence of human saliva. J. Med. Microbiol..

[27] Gittings S, Turnbull N, Henry B, Roberts CJ, Gershkovich P (2015). Characterisation of human saliva as a platform for oral dissolution medium development. Eur. J. Pharm. Biopharm..

[28] Steyer A, Marušić M, Kolenc M, Triglav T (2021). A throat lozenge with fixed combination of cetylpyridinium chloride and benzydamine hydrochloride has direct virucidal effect on SARS-CoV-2. COVID.

[29] Mao X (2020). Cetylpyridinium chloride: Mechanism of action, antimicrobial efficacy in biofilms, and potential risks of resistance. Antimicrob. Agents Chemother..

[30] Varga Z (2020). Electron microscopy of SARS-CoV-2: A challenging task: Authors' reply. Lancet.

[31] Chen M (2020). Elevated ACE-2 expression in the olfactory neuroepithelium: Implications for anosmia and upper respiratory SARS-CoV-2 entry and replication. Eur. Respir. J..

[32] Sasaki M (2021). SARS-CoV-2 variants with mutations at the S1/S2 cleavage site are generated in vitro during propagation in TMPRSS2-deficient cells. PLOS Pathog..

[33] Overbergh L (2005). Validation of real-time RT-PCR assays for mRNA quantification in baboons. Cytokine.

[34] Kishimoto M (2021). TMPRSS11D and TMPRSS13 activate the SARS-CoV-2 spike protein. Viruses.

