# Supplementary material for: Antiviral effect of cetylpyridinium chloride in mouthwash on SARS-CoV-2
Source: Sci Rep. 2022 Aug 18;12:14050. doi: 10.1038/s41598-022-18367-6 (PMC9386671; doi:10.1038/s41598-022-18367-6)
Supplement: Supplementary file 1 — Supplementary Figures. [file 41598_2022_18367_MOESM1_ESM.docx]

**Antiviral effect of cetylpyridinium chloride in mouthwash on SARS-CoV-2**

**Supplementary information**


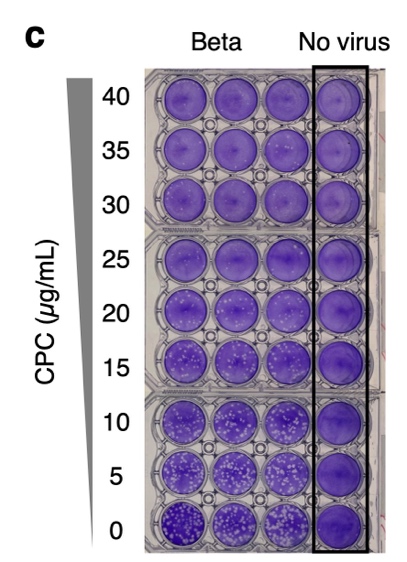

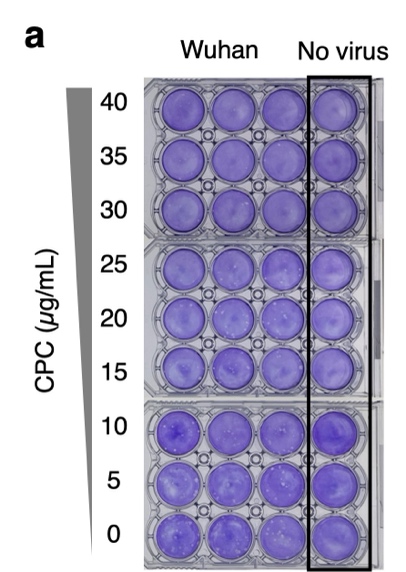

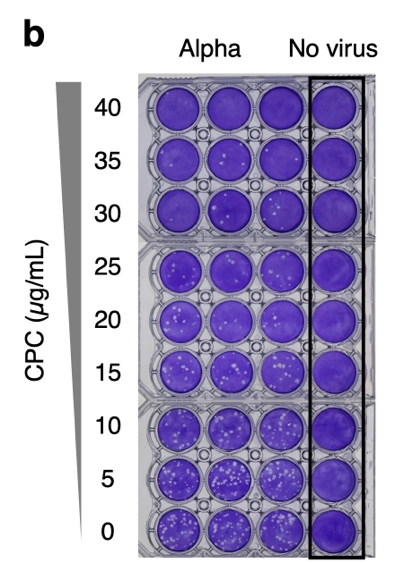

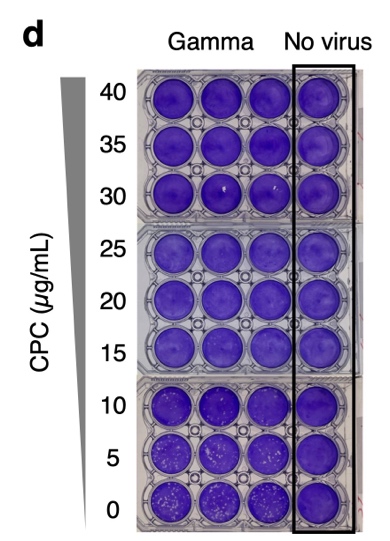


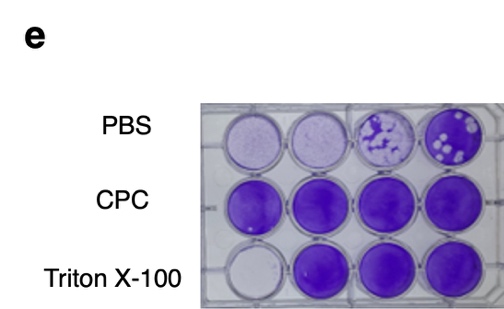


**Figure S1.** Antiviral efficacy of CPC against SARS-CoV-2 by plaque assay. The assay using Vero E6 cells expressing the TMPRSS2 gene (VeroE6/TMPRSS2). Plaque assay was performed using (**a**) Wuhan, (**b**) Alpha, (**c**) Beta and (**d**) Gamma strains treated with CPC (0-40 µg/mL) for 30 min at room temperature. Plaque assay was also performed in the presence of PBS, CPC (50 μg/mL) or Triton X-100 (1%) for 10 min. Thereafter, samples were filtrated by PD-10 columns to eliminate reagents (**e**).


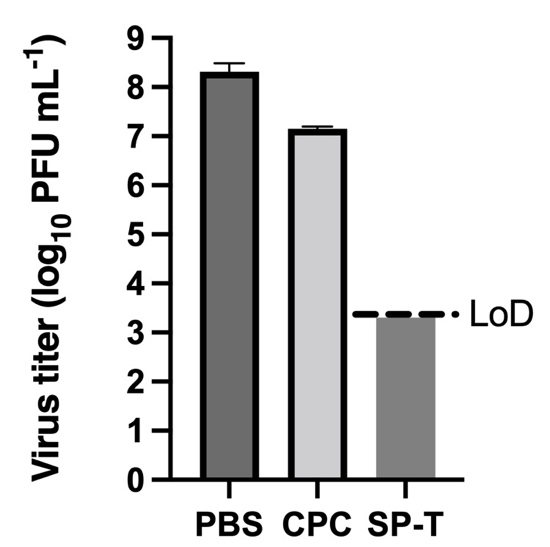


**Figure S2.** Antiviral efficacy of CPC and SP-T against SARS-CoV-2 by plaque assay. Plaque assay was performed using Wuhan strain treated with CPC (50 µg/mL) or SP-T (same concentration as 50 µg/mL CPC) for 30 min at room temperature. The virus titer of SARS-CoV-2 treated with SP-T was below the limit of detection (LoD) of 2.0 × 10^3^ PFU/mL.


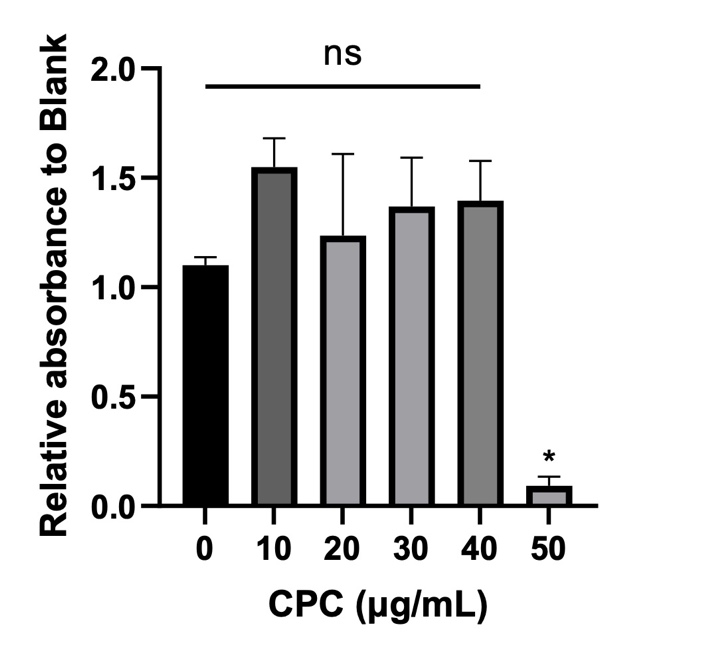


**Figure S3.** Cell survival assay by MTS assay. Cell viability of VeroE6/TMPRSS2 was measured by an MTS assay in the presence of different concentration of CPC (0-50 μg/mL) for 1 h at 37 °C. The absorbance was measured. Statistical analysis was performed using one-way analysis of variance. (**p* < 0.05)

**a b**


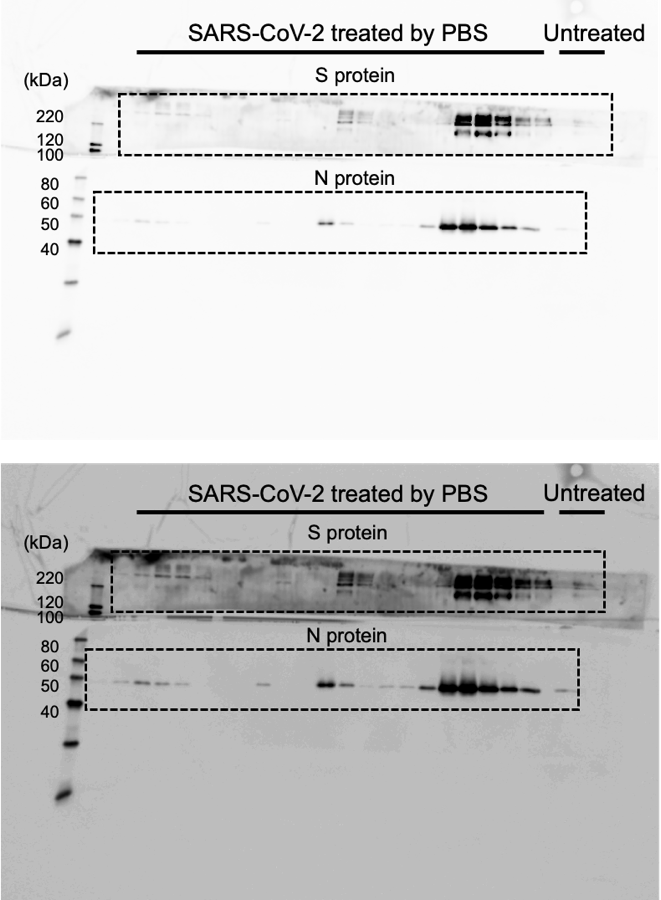

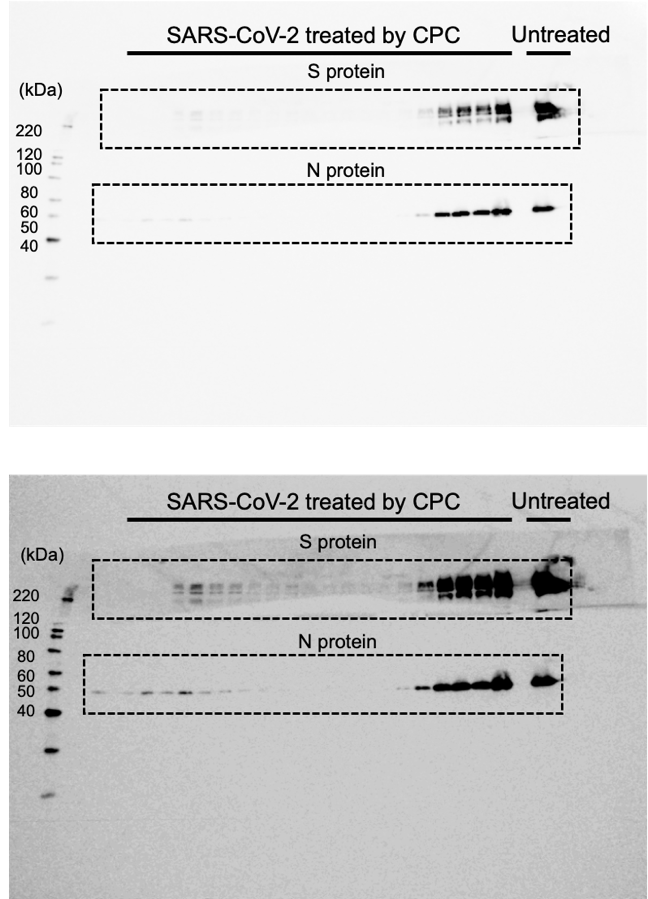


**c**


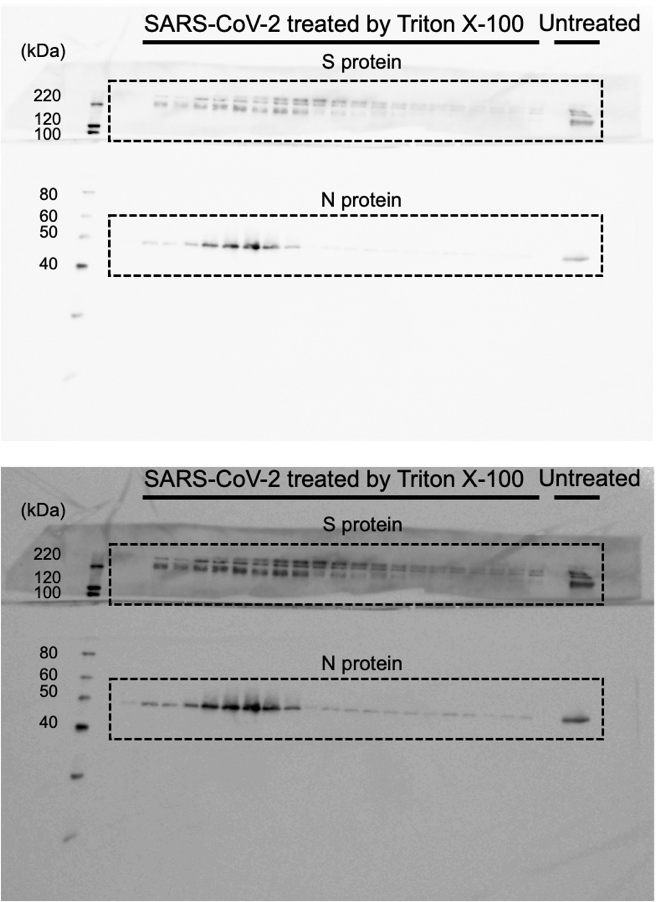
**Figure S4:** Uncropped western blots for Sucrose density analysis of SARS-CoV-2 particles. SARS-CoV-2 Wuhan strain was treated with 1× PBS (**a**), CPC (50 μg/mL) (**b**) and Triton X-100 (1%) (**c**) for 10 min, and the treated virions were applied to the density-gradient ultracentrifugation. Each fraction was applied to SDS-PAGE and analyzed by Western blotting with antibodies against S protein and N protein. The top images are the raw images, and the bottom images are the images in which exposure and contrast are varied.
